# The use of hyaluronic acid injection for treatment of black triangle and reconstruction of lost interdental papilla in anterior teeth: a systematic review

**DOI:** 10.2340/aos.v83.40864

**Published:** 2024-06-12

**Authors:** Shahad B. Alsharif, Bushra Aljahdali

**Affiliations:** Department of Periodontology, Faculty of Dentistry, King Abdulaziz University, Jeddah, Saudi Arabia

**Keywords:** Hyaluronic acid, injections, dental papilla, gingiva, dental aesthetics, teeth

## Abstract

**Objective:**

Non-surgical therapeutics to reconstruct lost interdental papilla are evolving; these include hyaluronic acid injection. The aim of this systematic review is to evaluate the efficacy, safety, and long-term outcomes of hyaluronic acid injection in the treatment of black triangles and reconstruction of lost interdental papilla in anterior teeth.

**Materials and Methods:**

The protocol was registered in PROSPERO (CRD42023446875) and in accordance with the Cochrane Handbook of Systematic Reviews of Interventions and the Preferred Reporting Items for Systematic Reviews and Meta-Analysis ‘PRISMA’. The search involved four databases, PubMed/MEDLINE, The Cochrane Library, Google Scholar, and ProQuest for ‘’grey literature’ with additional manual search for studies published up to May 2024. Human clinical studies of a prospective nature (randomised clinical trials and prospective cohort studies) were included. Exclusion criteria were case reports, case series, review articles, letter to editor, personal opinion, and animal studies. Furthermore, studies which utilised hyaluronic acid injection in conjunction with other therapeutic material, tissue graft, or any surgical procedure were also excluded. The data were extracted independently by the two authors and incorporated after consensus. The risk of bias was assessed using the RoB2: the revised Cochrane risk of bias tool for randomised clinical trials and the Newcastle Ottawa scale for prospective cohort studies.

**Results:**

24 studies, 15 prospective clinical studies and nine randomised clinical trials, were included with a total of 898 interdental papillae injected with hyaluronic acid. The studies showed promising outcomes in the reconstruction of lost interdental papilla with minimal adverse reactions. Risk of bias assessment among prospective clinical studies revealed 13 good quality studies with only two poor studies while the randomised clinical trials consisted of three with low, one with some concern, and five studies with high risk of bias. However, due to the high heterogeneity, a meta-analysis was not feasible.

**Conclusion:**

Hyaluronic acid injection is an effective minimally invasive approach in treating black triangles and reconstructing lost interdental papilla in the anterior teeth. Further long-term well-designed randomised clinical trials employing standardised procedures are essential to validate this treatment and provide better quality of evidence.

## Introduction

Patients’ demand for an aesthetic smile is growing as it affects their confidence and self-esteem. Both teeth as well as the gingival tissue are critical to obtain a satisfactory smile [1]. The interdental papilla is an anatomical part of the gingival tissue, which occupies the interdental spaces between the teeth termed ‘the embrasure’ [2]. Morphologically, it was described first by Cohen in 1959 [3]. Maintenance of the interdental papilla depends on the position of the adjacent teeth, the level of the proximal contact between teeth, and the level of the alveolar bone [4].

Interdental papilla loss creates open gingival embrasures, also called ‘black triangles’ [5]. Several factors influence the appearance of black triangles, these includes, age, attachment loss due to periodontal disease, abnormal teeth shape, root angulation, poorly contoured restorations and crowns, the presence of diastema, traumatic oral hygiene habits, thin gingival biotype, and the location of the interproximal contact, when the distance between the contact point and the crest of the bone is ≥5 mm [4–11].

Although the interdental papilla is a small anatomical component, it is of major aesthetic significance, especially in the anterior teeth [12]. Black triangles were found to be the third most dislikable aesthetic issue affecting an individual’s smile, as ranked by patients, following dental caries and improper crown margins [13]. In addition to the aesthetic concern, black triangles can contribute to phonetics difficulties as reported by patients [14]. Therefore, treatment of black triangles is crucial to restore function and aesthetic. However, reconstruction of lost interdental papilla remains a challenging procedure with unpredictable long-term results.

Several periodontal plastic surgical approaches were proposed in attempt to re-create the lost interdental gingival tissue; these utilised different flap designs and various grafting materials [15–21]. However, these surgical methods are not only considered invasive procedures but also their effectiveness and outcome remain unpredictable. This could be attributed to the fact that interdental papilla is a small and narrow structure, which complicates the surgical procedure because of access difficulties. Additionally, the limited blood supply in the area is a further challenge, which ultimately affects the healing process [22]. Due to these concerns, researchers have been seeking other less-invasive non-surgical treatment alternatives to reconstruct lost interdental papilla.

These non-surgical approaches include hemolasertherapy [23], orthodontic treatment [24], restorative treatment [25], and the injection of fillers and gingival tissue volumisers [26–29], either solitary or in combination with other approach [30]. Different injectable materials have been proposed for papillary reconstruction in recent studies, these include hyaluronic acid [28], platelet rich plasma [27], stem cells [29], and fibroblasts [26]. This less-invasive approach was found to be a safe promising procedure and less traumatic compared to the surgical treatment with immediate results obvious to the patients right after the injection [31].

Hyaluronic acid is a natural polysaccharide, an anionic non-sulphated glycosaminoglycan; it is a main component of extracellular matrix and present widely in different body tissues including cartilage, skin, neural tissue, and gingival tissue [32, 33]. It is frequently used in dermatology as filler material and skin moisturiser due to its hygroscopic nature. It has the ability to absorb water, swell the tissue, thus, resulting in smoother and fuller contour [34, 35]. In addition, it can be used as space-filling material in the treatment of black triangles because of papilla loss [33], contribute positively to wound healing process [36], and maintain tissue haemostasis and structural integrity [37].

Possible reconstruction of lost interdental papilla using hyaluronic acid injection was proposed previously [38]. This minimally invasive and relatively inexpensive technique is showing great interest as studies utilising this non-surgical approach showed promising results [39, 40]. However, its routine clinical use remains questionable. There is still a concern regarding the predictability, the long-term outcome, and tissue stability of this technique. In addition, adverse effects associated with this technique were reported, including lip tenderness, swelling, and burning sensation [41].

Therefore, the aim of this systematic review is to summarise the existing evidence regarding the efficacy, safety, and long-term outcomes of the use of hyaluronic acid injection for reconstruction of lost anterior interdental papilla as no consensus has been established up to now.

## Materials and methods

### Protocol registration

The protocol of this systematic review was registered in the International Prospective Register of Systematic Reviews ‘PROSPERO’, the National Institute for Health Research, University of York, UK, Center for Reviews and Dissemination; the registration ID number is CRD42023446875. The protocol of this systematic review was in accordance with the Cochrane Handbook of Systematic Reviews of Interventions [42] and the Preferred Reporting Items for Systematic Reviews and Meta-Analysis ‘PRISMA’ [43].

### PICO question

The systematic review was conducted following PRISMA guidelines [44] with Patient, problem, or population; Intervention; Comparison, control, or comparator; Outcome (PICO) question formulated as follows: ‘Is hyaluronic acid injection effective for reconstruction of lost interdental papilla in adult patients?’

– Population: Adult patients with lost interdental papilla (black triangles) in the anterior teeth.
– Intervention: Hyaluronic acid injection.
– Comparison: The changes in the interdental papilla volume before and after hyaluronic acid injection in comparison to control (no treatment), if available, or other materials injected.
– Outcome: The reconstruction of lost interdental papilla measured by black triangle height (BTH), black triangle area (BTA), interdental papilla height (IDPH), papillary presence index (PPI), black triangle width (BTW), complete interdental papilla reconstruction (CIPR), partial interdental papilla reconstruction (PIPR), height of gingival papilla (HGP), interdental papillary defect size (IDPDS), papilla reconstruction percentage (PR), deficient papilla percentage (DP), contact point to gingival margin distance (CP-GM), interproximal width (IPW), papilla gain oscillation (PGO), and papilla tip to contact point distance (PT-CP).

### Inclusion and exclusion criteria

Studies included in this review were human clinical studies of a prospective nature (randomised clinical trials and prospective cohort studies), published in English language, reporting before and after outcomes of hyaluronic acid injections used for papilla reconstruction in the anterior teeth performed in adult patients. No restrictions regarding age groups, number of papillae treated, the time of follow up, or years of publication were applied.

Exclusion criteria included case reports, case series, review articles, letter to editor, personal opinion, animal studies, and articles published in languages other than English. In addition, studies which utilised hyaluronic acid injection in conjunction with other therapeutic material, tissue graft, or any surgical procedure as the outcome reported might not be due to the effect of hyaluronic acid injection exclusively; rather, it could be a combination effect or because of the effect of the other combined approach with minimal or no effect of hyaluronic acid injection itself.

### Source of information and search strategy

Comprehensive literature search was performed by two independent reviewers, the authors (S.B.A. and B.A.), with no publication time restriction (no starting date) up to May 2024. The search included the following electronic databases, PubMed/MEDLINE, The Cochrane Library, and Google Scholar. Additional search for ‘grey literature’ was performed in the ProQuest database. The harvest was further complemented by a manual search of the references list of the selected articles. The Medical Subject Heading (MeSH) terms and keywords were designated based on the PICO of this systematic review with the use of ‘AND’ and ‘OR’ customised for each database. Detailed search strategies for each database are presented in Table 1.

**Table 1:** Detailed search strategy for each database.

Database	Search strategy
PubMed/MEDLINE	((interdental papilla OR papilla OR interproximal gingiva OR gingiva OR interdental papilla defect OR interproximal gingival defect OR black triangles OR missing papilla OR papilla loss OR deficient papilla OR papilla reconstruction OR papillary recession OR papilla augmentation[MeSH Terms]) AND (hyaluronic acid OR HA OR hyaluronic acid gel OR hyaluronic acid injection OR hyaluronan OR HY OR hyaluronan injection OR hyaluronate OR hyaluronate injection[MeSH Terms])) AND (adults OR patients OR humans[MeSH Terms])
The Cochrane Library	(interdental papilla OR papilla OR interproximal gingiva OR gingiva OR interdental papilla defect OR interproximal gingival defect OR black triangles OR missing papilla OR papilla loss OR deficient papilla OR papilla reconstruction OR papillary recession OR papilla augmentation) AND (hyaluronic acid OR HA OR hyaluronic acid gel OR hyaluronic acid injection OR hyaluronan OR HY OR hyaluronan injection OR hyaluronate OR hyaluronate injection) AND (adults OR patients OR humans)
Google scholar	(‘dental papilla’ OR ‘interdental papilla’ OR ‘interproximal papilla’ OR ‘black triangles’ OR ‘papillary recession’) AND (‘hyaluronic acid’ OR ‘hyaluronan’ OR ‘HA’ OR ‘HY’ OR ‘hyaluronate’) AND (‘patients’ OR ‘humans’)
ProQuest	(‘dental papilla’ OR ‘interdental papilla’ OR ‘black triangles’) AND (‘hyaluronic acid’ OR ‘hyaluronan’ OR ‘hyaluronate’) AND (‘patients’ OR ‘humans’)

### Studies selection

**Figure 1:**
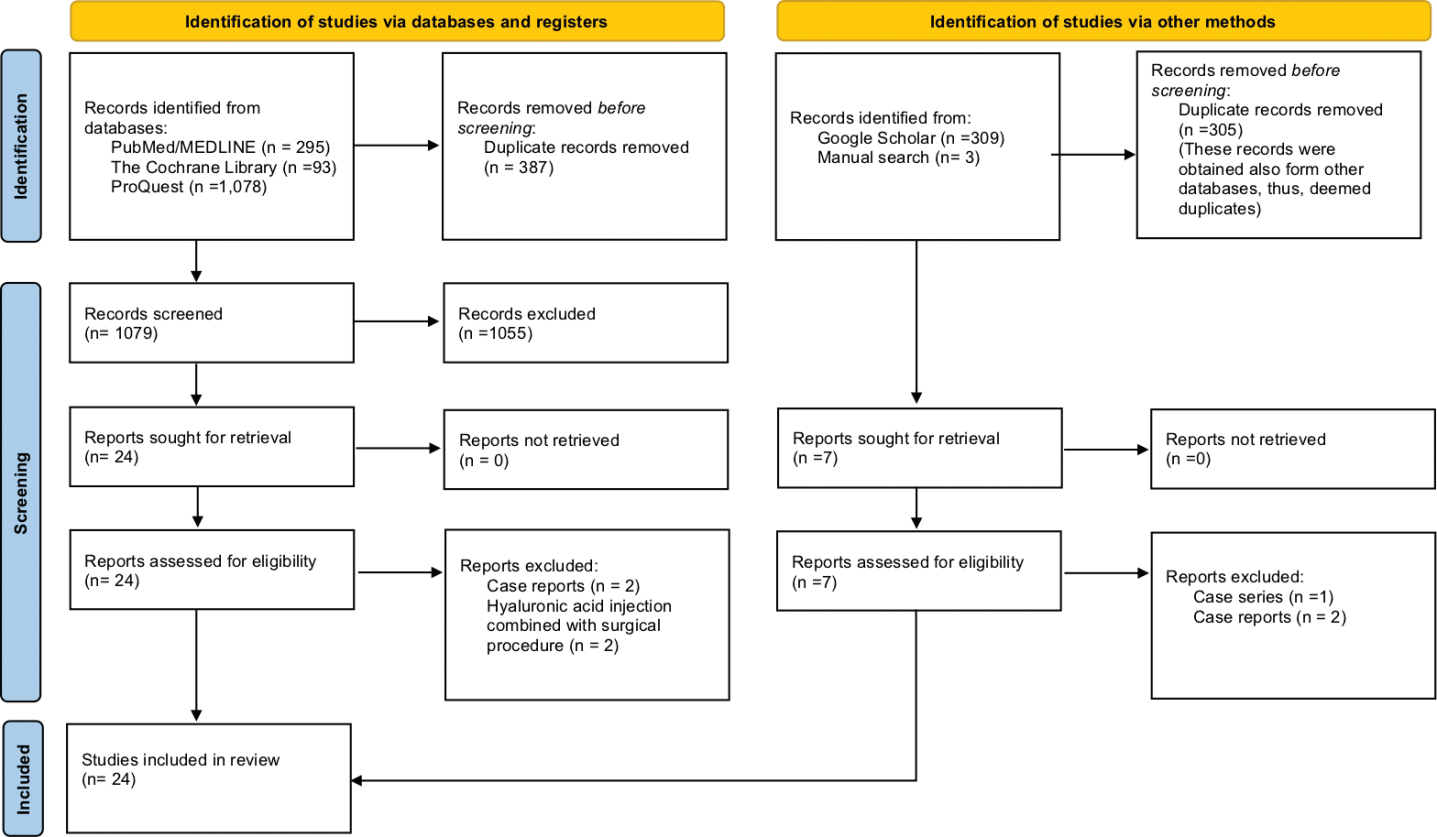
PRISMA flowchart with search details and reason for exclusion.

Studies selection and eligibility assessment was performed by the same two independent reviewers (S.B.A and B.A.), based on the customised inclusion and exclusion criteria in the following stages: First: the obtained search studies were exported to Microsoft Excel (Microsoft Ltd, Washington) in which duplicates were removed. Second: reviewers’ calibration assessment was performed on part of the retrieved search to calculate inter-rater agreement on studies selection using Cohen’s kappa statistic, which was found to be substantial (k = 0.74) [45]. Third: after calibration, the two reviewers independently conducted the initial search by assessing the titles and abstracts for all studies. If both reviewers agreed on the exclusion, the study was excluded. However, in case of disagreement, the full manuscript was read to get a final decision. Fourth: Full manuscripts were evaluated to confirm the eligibility criteria for inclusion, thus, to exclude studies that were judged ineligible. The reason for exclusion was clarified in Figure 1 and Table 2. At the final stage, if there was disagreement between the two reviewers, a third reviewer was consulted for the final decision.

**Table 2:** List of the excluded studies and the reason for exclusion.

The excluded study	Reason for exclusion
Neto ADT, Storrer CLM, Santos FR, Miran T. Increased papilla between implant and tooth with TEH use of hyaluronic acid injection: A case report. IP Int J Periodontol Implantol. 2018;3:24–9.	Case report + combined treatment
Spano SJ, Ghilzon R, Lam DK, Goldberg MB, Tenenbaum HC. Subperiosteal Papilla Augmentation With a Non-Animal-Derived Hyaluronic Acid Overlay Technique. Clin Adv Periodontics. 2020;10:4–9.	Hyaluronic acid injection combined with surgical procedure
Naorungroj S. Esthetic Reconstruction of Diastema with Adhesive Tooth-Colored Restorations and Hyaluronic Acid Fillers. Case Rep Dent. 2017;2017:5670582.	Case report + combined treatment
Tanwar J, Hungund SA. Hyaluronic acid: Hope of light to black triangles. J Int Soc Prev Community Dent. 2016;6:497–500.	Case report
Hamadeh W, Alhabashneh R, Abdelhafez R, Khader Y. Management of interdental papillary defects using subperiosteal hyaluronic acid injection overlay technique: a prospective longitudinal clinical study. Quintessence Int. 2024;55:180–9.	Hyaluronic acid injection combined with surgical procedure
Lestari V, Nasution AH. The use of hyaluronic acid dermal filler in black triangle cases management: A case series. Bali Medical Journal. 2024;13:1–88.	Case series
Sánchez D, Yáñez Ocampo B, Esquivel Chirino C. Use of hyaluronic acid as an alternative for reconstruction of interdental papilla. Rev. Odont. Mex. 2017;21:205–13.	Case report

### Data extraction

The same two reviewers (S.B.A and B.A.), performed the data extraction independently, afterward, it was compared for precision. In case of any inconsistencies, the two reviewers had a discussion until consensus was reached. Full manuscripts were read and inspected for the following data: author, year of publication, location where the study was performed, study design, number of patients, patients dropouts, age, gender, inclusion criteria, exclusion criteria, number of injected interdental papillae, location: maxilla or mandible, classification type of papilla loss, history of periodontal treatment before hyaluronic acid injection, details regarding hyaluronic acid used: type, commercial brand, concentration, and volume injected, location of the point of injection and injection technique, injection protocol: single or multiple, if multiple: intervals or time between sessions, post procedure instruction, papilla measurement tool, follow up intervals, details of outcomes measured, post procedure complications, patient satisfaction, and the final main conclusion. No assumption was made regarding any missing information; instead, ‘not applicable’ was stated.

### Risk of bias in individual studies

To determine the risk of bias in the selected articles, the same two reviewers (S.B.A and B.A.), independently performed the evaluation and discrepancy was discussed until consensus was reached. For randomised clinical trials, the RoB2: the revised Cochrane risk of bias tool for randomised trials was followed, which is based on five domains: D1: randomisation process, D2: deviations from intended interventions, D3: missing outcome data, D4: measurement of the outcome, D5: selection of the reported result. Low, some concern, or high risk of bias to be assigned to each domain, then, an overall risk of bias will be allocated [46]. For prospective clinical studies, the Newcastle Ottawa scale for prospective cohort studies was implemented, which is based on three domains: selection (maximum 4 stars), comparability (maximum 2 stars), and outcome (maximum of 3 stars). Then, based on the obtained total number of stars, each study is assigned to good, fair, or poor quality [47].

## Results

### Search results

Considering the mentioned protocol and search strategy presented in Table 1, a total of 1778 articles were obtained from the different databases, PubMed/MEDLINE (*n* = 295), The Cochrane library (*n* = 93), ProQuest (*n* = 1,078), Google Scholar (*n* = 309), and manual search (*n* = 3). After eradicating the duplicates (*n* = 692), screening the titles and abstracts according to the eligibility criteria 31 articles were yielded for full-text reading. After further evaluation of the full manuscripts, a total of 24 studies were included in this systematic review. Details on the search strategy and reason of exclusion are illustrated in Figure 1 and Table 2.

### Characteristics of included studies

Of these 24 studies included, 15 were prospective clinical studies [39, 40, 48–60], and nine were randomised clinical trials [61–69]. Table 3 summarises the characteristics of the included studies. Total of 898 interdental papillae were injected with hyaluronic acid, all in the anterior area and mostly maxillary teeth. Most studies investigated the effect of hyaluronic acid injection in treatment of lost interdental papilla prospectively over time [39, 40, 48–60, 62]. One study compared hyaluronic acid injection with negative control [64], three studies considered comparison with placebo control (saline injection) [61, 65, 67], while four studies compared hyaluronic acid injection to different materials, these include, plasma rich growth factors (PRGF) [63], injectable platelet rich fibrin (iPRF) [68], calcium hydroxyapatite filler (Radiesse) [69], and chitosan [66]. Different commercial brands of hyaluronic acid were utilised.

**Table 3:** Characteristics of the included studies.

Author, Year	Country of study	Study design	Patients	Gender	Age (years)	Inclusion criteria	Exclusion criteria
Turgut Çankaya and Tamam, 2020 [48]	Turkey	PCS	20	10 F 10 M	24–44 mean 34	Open interdental embrasure in natural teeth, no systemic disease, non-smokers, not pregnant or lactating females, no diagnosis of periodontitis, no use of antibiotics or any drugs affecting the periodontal tissues in the last 3 months, absence of prosthetic restoration in the maxillary and mandibular anterior teeth, five interdental papillary spaces in the inter canine–canine region	No contact point, insufficient plaque control, pocket depth > 3 mm, < 2 mm keratinised gingiva
Abdelraouf et al., 2019 [61]	Egypt	RCT	10, 2 dropouts	7 F 3 M	21–47 mean 32.55 ± 9.3	Highly motivated patients, at least one deficient papilla in the inter-bicuspid region, papillary deficiency types I or II (Nordland and Tarnow), distance between the contact point and inter-proximal bone crest ≤ 7 mm, probing depth ≤ 4 mm at the deficient papillary sites, full mouth plaque index and gingival index scores 0–1, no open contacts between affected teeth, no caries, no proximal restorations, no fixed prosthesis or orthodontic appliances	Subjects with medical conditions affecting periodontal healing or regeneration, a history of allergic reactions, pregnant or breastfeeding females, smokers, alcoholics, current or previous drugs intake that may predispose to gingival enlargement, under orthodontic treatment or had it in the past 6 months, history of traumatic oral hygiene measures or periodontal surgeries over the last 6 months at the area of interest
Bertl et al., 2017 [62]	Denmark	RCT	22, 1 dropout	12 F 9 M	HA: mean 26.7 ± 5.2 Saline: mean 33.1 ± 6.0	≥ 18 years of age, implant restoration finalised > 6 months ago, at least one deficient papilla in the anterior maxilla (first premolar to first premolar) between a natural tooth and an implant-supported crown	No contact point, inadequate plaque control (full-mouth plaque score > 20%), probing depth > 5 mm, buccal gingival recession > 3 mm, < 2 mm keratinised tissue at the adjacent tooth/ implant, systemic disorders, regular intake of medications affecting connective tissue metabolism
Bal et al., 2023 [63]	India	RCT	21, 2 dropouts	9 F 10 M	18–45 (HA: mean 34.63 ± 5.22, HA+ PRGF: mean 38.71 ± 8.4)	Patients with aesthetic concern, food lodgement in the anterior embrasure, Class I and Class II papillary loss (Nordland and Tarnow), adequate width of attached gingiva, age 18–45 years, plaque index < 1 (Turesky, Gilmore and Glickman Modification of Quigley Hein); gingival index < 1 (Loe and Silness), distance ≤ 7 mm from the interdental contact point to the interproximal bone crest, probing depth ≤ 4 mm at the defective papillary sites	Patients who received radiotherapy, chemotherapy, immunosuppressive treatments, corticosteroids, anticoagulants the 30 days prior to intervention, history of allergy, systemic or blood borne diseases, prolonged treatment with non-steroidal anti-inflammatory drugs or similar medications, smokers, lactating or pregnant females, presence of composite and prosthetic restoration in maxillary anterior region, undergoing orthodontic treatment, having high frenum attachment, having midline diastema, having any inability to take part in the investigation and comply with the required follow-up procedures, sites with Class III papillary loss (Nordland and Tarnow), sites with underlining intraosseous defects and implant sites
Awartani and Takis, 2016 [49]	Saudi Arabia	PCS	10, 1 dropout	9 F	22– 55 average 36.4	Adults ≥18 years old, systemically healthy, at least one maxillary or mandibular anterior interdental space exhibiting class I or II interdental papilla loss (Nordland and Tarnow)	History of allergic reaction to injectable filler, smoking, pregnancy and lactation, medications affecting the gingiva or wound healing, periodontal surgery in the last 12 months, carious lesions or fixed restorations on the study teeth, periodontitis, poor plaque control, visible plaque present, full mouth plaque score > 20 % (O’Leary)
da Silva et al., 2023 [51]	Brazil	PCS	6	2 F 4 M	NR	Presence of defective tissue in the interdental papillae in the aesthetic areas between teeth, between teeth and implants, and/or between implants	Significant systemic diseases, active periodontal diseases, smokers
Alhabashneh et al., 2021 [52]	Jordan	PCS	27, 6 dropouts	14 F 7 M	mean 36.9 ± 9.16	Caucasian, nonsmokers, age > 18 years, at least one site with interdental papilla recession in the anterior region of the maxillary or mandibular jaws, Class I or Class II papillary recession (Nordland and Tarnow), distance from the contact point to alveolar bone crest ≥5 mm, no active periodontal diseases, good oral hygiene	Spacing or crowding between the teeth to be treated, abnormal tooth shape, systemic diseases such as diabetes mellitus, hypertension or conditions that alter the outcome of periodontal therapy, pregnant, lactating women, tobacco users
Pitale et al., 2021 [53]	India	PCS	7	5 F 2 M	mean 30.96	Healthy patients, no radiographic evidence of interdental bone loss, pocket depth ≤ 4 mm, good plaque control, no tooth mobility	Systemic disease, blood disorders, pregnant or lactating mothers, tobacco users
Becker et al., 2010 [39]	USA	PCS	11	7 F 4 M	25–75 average 55.8	Deficient papillae adjacent to teeth or implants	
Patel et al., 2017 [54]	India	PCS	4	3 F 2 M	20–65	Systemically healthy adults, at least one anterior site with class I or class II interdental papilla loss, aesthetics as their chief concern, age 20–65 years, maxillary anterior teeth, plaque index < 20%, no caries or prosthesis, non-smokers, no systemic diseases affecting the periodontium or drugs causing gingival overgrowth	
Mandel et al., 2020 [64]	Hungary	RCT	40, 9 dropouts	23 F 8 M	18–70 average 44.4 ± 12.8 (Revident: mean 46.1 ± 12.3, Flex Barrier: mean 41.8 ± 13.8)	Age 18–70, at least two upper and two lower interdental papillary defects in the front region between canine teeth, Class I or Class II papillary defects (Nordland & Tarnow)	Active periodontitis, acute oral and/or upper respiratory tract infection, previous surgical treatment of the papillae to be investigated, pregnancy or lactation, smoking, bleeding disorders or any medication that would affect blood coagulation, systemic diseases affecting the periodontal health, any kind of ongoing immunosuppression, known or suspected allergy to local anaesthetics and/or hyaluronic acid
Ni et al., 2019 [56]	China	PCS	8	8 F	28–60 average 41.6	Adults 20–60 years, no systemic disease, good oral hygiene (full mouth plaque score< 20%), at least one interdental papilla loss in the anterior maxilla or mandible, healthy periodontal tissue or the inflammation is controlled	Fixed prosthesis or carries, no contact point, history of allergic reaction to injectable filler, periodontal surgery in the last 6 months, drugs affecting the gingiva metabolism
Ni et al., 2021 [65]	China	RCT	24, 3 dropouts	19 F 2 M	28–63 average 41.3 ± 7.73	Adults 20–70 years, no systemic disease such as hypertension, coronary artery disease, stroke, or type II diabetes, no fixed prostheses or caries, good oral hygiene (full- mouth plaque score < 20%), 2 or 4 symmetrical gingival papilla defects (Class I or Class II, Nordland & Tarnow) in the anterior maxilla, healthy periodontal tissue or well controlled inflammation (< 10% bleeding sites, probing depths ≤ 3 mm), no history of periodontal surgery in the last 6 months, non-smokers, no history of drugs affecting gingiva metabolism such as calcium channel blockers or cyclosporin A	
Patil et al., 2020 [58]	India	PCS	8	5 F 3 M	27–35 mean 32	At least one papillary deficient site in the upper anterior area, plaque index and gingival index 0-1	Pregnancy, patients taking medication causing gingival enlargement, currently receiving orthodontic treatment on the upper anterior area
Kapoor and Bhardwaj, 2022 [67]	India	RCT	15	NR	20–61 mean 37.5 ± 14.4 (HA: mean 37.13, Saline: mean 33.4)	Possession of the maxillary anterior teeth, age 20–61 years, at least one papillary deficient site in the upper anterior area, non-smoker, non-contributory medical history, no consumption of drugs causing gingival hyperplasia	Pregnancy and lactation, history of allergy to hyaluronic acid gel, parafunctional habits, currently receiving orthodontic treatment on the upper anterior area
Fakher et al., 2023 [68]	Egypt	RCT	NR	NR	NR	Age 20–50 years, highly motivated patients with no missing teeth in the anterior region having deficient papilla class I and II (Nordland and Tarnow), papillary marginal gingival index = 0, no open contacts between the affected teeth, no systemic disease	Smokers, pregnancy and lactation, on medications known to cause gingival enlargement, history of gingival fillers, caries, proximal restorations, fixed or orthodontic appliances, periodontal surgeries 6 months prior
Lee et al., 2016 [59]	South Korea	PCS	10	6 F 4 M	27–35 mean 32	Adult patients, at least one interdental papilla deficiency with the presence of a contact point between adjacent teeth in the maxillary anterior region, plaque and gingival indices 0–1	Pregnancy, on medication known to cause gingival hyperplasia, currently receiving orthodontic treatment in the maxillary anterior region
Lee et al., 2016 [57]	South Korea	PCS	13	7 F 6 M	27–35 mean 32	At least one papillary deficient site in the upper anterior area, plaque index and gingival index 0-1	Pregnancy, on medication known to cause gingival enlargement, currently receiving orthodontic treatment on the upper anterior area
Singh and Vandana, 2019 [55]	India	PCS	10	NR	25–40 mean age range 29.6–30.6	Systemically and periodontally healthy, clinically normal periodontium other than deficient papilla with Cardaropoli Papilla Presence Index score 2 and 3, full mouth plaque score < 10%	Allergy to hyaluronic acid, poor plaque control, medically compromised, teeth with hopeless prognosis, parafunctional habits, traumatic occlusion, had periodontal plastic surgery for the selected area in the last 1 year, adjacent teeth with caries, fixed prosthesis, or orthodontic appliance, drug-induced gingival overgrowth, pregnancy and lactation, smokers
Sadat Mansouri et al., 2013 [40]	Iran	PCS	11	8 F 3 M	22–61 mean 37.5 ± 14.4	Age 20–75 years, possession of the maxillary anterior teeth, plaque index < 20%, no caries, no fixed prosthesis or orthodontic appliance, non-smokers, no history of systemic disease affecting the periodontal health, no consumption of drugs causing gingival hyperplasia	
Shawky and Darwish, 2017 [69]	Egypt	RCT	30	30 F	NR	Age 25–35 years, plaque index < 20%, papillary marginal gingival index = 0, probing depth < 3 mm, clinical attachment loss 0–2 mm, no caries, no fixed prosthesis or orthodontic appliance	Patients with systemic disease, on drugs causing gingival hyperplasia, smokers, pregnancy and lactation
Abdelkader and Ebrahem, 2022 [66]	Egypt	RCT	NR	NR	20–45	At least one deficient papilla in inter- bicuspid region interdental space exhibiting class 1 or 2 (Tarnow), no systemic diseases that might influence the periodontal health, no regenerative periodontal therapy 6 months before the initial examination	Pregnancy and lactation, smokers
Firkova 2020 [50]	Bulgaria	PCS	19	NR	23–72	Non-smokers, no uncontrolled systemic diseases or drugs which could affect gingival condition, maxillary anterior teeth, no caries lesions, restorations, veneers or prosthetic crowns, orthodontic appliances, plaque index < 15%, papillary deficiency, no diastema, distance between the contact point and alveolar bone crest ≤ 5 mm	Medical conditions affecting the periodontal healing, drug intake affecting the gingiva, history of allergy or periodontal surgeries in the past 6 months
Ebrahimi et al., 2023 [60]	Iran	PCS	4	NR	NR	Interdental papilla loss in the anterior region of jaws (classes I and II Nordland and Tarnow), no gingivitis or periodontitis, and a plaque index of less than 20%, no active periodontal disease, no bleeding on probing, no pocket depth > 5 mm, no tooth mobility	Smoking, systemic diseases that might affect the periodontal health or scar healing, medications causing gingival enlargement, teeth crowding, caries, calculus, or wearing orthodontic appliance, history of allergy to injectable fillers, pregnancy and breastfeeding, history of periodontal surgery in the past 12 months

RCT: Randomised clinical trial; PCS: Prospective clinical study; NR: Not reported; F: Females; M: Males; HA: Hyaluronic acid; PRGF: Plasma rich growth factors; mm: millimetres.

Baseline deficient interdental papillae were evaluated and classified based on Nordland and Tarnow classification [10] in 15 studies [49, 50, 52–54, 56, 58, 60, 61, 63–68], the modified papillae index score (MPIS) [70] in two studies [48, 62], Cardaropoli classification [71] in one study [55], while six studies did not specify their papillae classification system [39, 40, 51, 57, 59, 69]. Initial non-surgical periodontal treatment was described in most of the studies [40, 48–50, 52, 53, 56, 61, 63–67]. Generally, it included patient motivation, oral hygiene instructions, and full mouth debridement. Injection technique of hyaluronic acid varied among the included studies; mostly, 0.2 ml of hyaluronic acid gel was injected, five studies performed single injection [53, 63, 64, 66, 69], another five studies repeated the injection process twice [49, 50, 52, 54, 62], while the majority utilised multiple injections protocol from 3 and up to 5 times [39, 40, 48, 51, 55–61, 65, 67, 68]. The location of the injection was 2–3 mm apical to the coronal tip of the deficient papilla at 45-degree angle in most studies. Post injection instructions were reported, mainly to avoid mechanical plaque control within the first 24 h at the treatment area. Details of the total number of injected papillae, region, hyaluronic acid used, injection protocol, pre-injection periodontal treatment, injection location and procedure, and post injection instructions are reported in Table 4.

**Table 4:** Details of hyaluronic acid injection procedures.

Author, Year	Injected papillae	Region (Max, Mand)	Details of HA used	Injection protocol	Pre-injection periodontal treatment	Injection location and procedure	Post-injection instructions
Turgut Çankaya and Tamam, 2020 [48]	200	Both	2 mg/mL HA non-cross-linked and 16 mg/mL HA cross-linked commercially available HA gel, hyaDENT BG, BioScience	Multiple injections if needed at 3 weeks intervals	Oral hygiene instructions, tooth surface cleaning with an ultrasonic scaler, when necessary, root scaling and planning under local anaesthesia	The injection points were determined as an imaginary triangle, with the peak point of the papilla forming the top of the triangle and the line drawn joining the peak points of the adjacent teeth forming the base of the triangle, three equidistant injections were made to the region corresponding to the triangle base; one injection was made to the top of the triangle, and two injections were made to the area between these two points, the needle was slowly withdrawn while the injection was continued	Control plaque normally in all the teeth except for interdental flossing in the treated area, which was continued after the second session
Abdelraouf et al., 2019 [61]	30 (HA:16, Saline: 14)	NR	0.1 mm of HA gel, Restylane Lidocaine cross-linked HA Filler, Galderma S.A, Sweden, or saline with concentration of 20 mg/m	Multiple injections at baseline, 3 weeks and 6 weeks	Full mouth supragingival scaling and subgingival debridement, patient motivation and education for proper oral hygiene instructions	The needle was inserted 2–3 mm apical to the tip of the interdental papilla and directed coronally with an angulation of 45° to the long axis of the tooth, and the bevel directed apically, the papilla was lightly massaged in an incisal direction for 1 min using gauze	24 h abstinence from mechanical plaque control in the area, followed by soft toothbrush, the use of mouthwash twice daily, routine mechanical oral hygiene was resumed after 2 weeks
Bertl et al., 2017 [62]	21	Max	0.2 ml of .08% HA gel, Hyadent Barrier Gel, BioScience, Ransbach- Baumbach, Germany; 1 ml contains 16 mg cross-linked Na-hyaluronate and 2 mg Na-hyaluronate	Twice at 4 weeks interval	NR	Three-step injection technique: creation of a reservoir in the mucosa immediately above the mucogingival junction, injection into the attached gingiva/mucosa just below the base of the deficient papilla, injection 2–3 mm apically to the tip of the deficient papilla	NR
Bal et al., 2023 [63]	34 (HA:17, HA+ PRGF: 17)	Max	0.2 mL of 0.8% HA gel, Gengigel®, Ricerfarma s.r.l., Milano, Italy	Single injection	Full mouth supragingival and subgingival instrumentation under local anaesthesia with ultrasonic instrument and periodontal Gracey’s Curettes if needed, oral hygiene instructions	2–3 mm apical to the tip of the interdental papilla and oriented coronally with 45° angulation with respect to the tooth’s axis, and the bevel was directed apically, the papilla was gently massaged for 1 min with digital pressure in an incisal direction using a gauze	Not performing mechanical plaque control in the area for 24 h with mouthwash (0.2% chlorhexidine digluconate) twice daily, followed by soft toothbrush after 24 h with the mouthwash
Awartani and Takis, 2016 [49]	17	Both	0.2 ml of the cross-linked HA clear gel	Twice at 4 weeks interval	Oral hygiene instructions, when necessary, supragingival debridement	Directly into the middle of the papilla, 2–3 mm apical to the tip of the papilla, followed by gentle massage of the area for 1 min	24 h abstinence from mechanical plaque control in the area, the use of soft toothbrush after the first 24 h, resumption of routine mechanical oral hygiene after 2 weeks
da Silva et al., 2023 [51]	19	NR	< 0.2 ml of 23 mg/ml Rennova HA gel (Innovapharma), monophasic, doubly sterilised, low cross-linking, viscoelastic (variable dynamic viscosity: 300,000–400,000 MPa).	Multiple injections, 3 injections at 4 weeks intervals	NR	2–3 mm above the papilla tip at a 45° angle in relation to the bone plate	NR
Alhabashneh et al., 2021 [52]	86	Both	0.2 ml of HA (HYADENT BG)®	Twice, repeated once after 21 days	Phase I periodontal therapy 4 weeks before HA injection, including supragingival and subgingival scaling, using ultrasonic scaler	Needle was inserted at a 45° angle with the bevel directed towards the bone	Keep good oral hygiene, brush regularly using a soft toothbrush, use an antiseptic mouthwash twice daily, free to take any non- prescription analgesic as required
Pitale et al., 2021 [53]	25	NR	0.2 ml of pure cross-linked mild type, 20 mg/ml concentrated, 400 μm (particle size), GENOSS® (MONALISA, Genoss Co. Ltd., Gyeonggi R&DB Center IF, Suwon-si Yeongtong-gu, Gyeonggi-do, Korea	Single injection	Thorough plaque control program was initiated 3 weeks before procedure, including oral hygiene instruction, patient education, and motivation	2–3 mm apical to the coronal tip of papillae at a 45° angle, the area was gently massaged for 2–3 min to ensure the uniform distribution of the filler	Soft diet, not to attempt to incise with their anterior teeth, a periodontal maintenance program weekly for the first 4 weeks then monthly until the end of the study period
Becker et al., 2010 [39]	14	NR	< 0.2 ml of a commercially available, hyaluronic-based gel	Multiple injections repeated up to 3 times with 3 weeks intervals	NR	2–3 mm apical to the coronal tip of the involved papillae	NR
Patel et al., 2017 [54]	8	Max	~0.2 ml injectable HA gel (non-animal based)	Twice, repeated once after 20 days	NR	The base of papilla 2–3 mm apical to the coronal tip of the involved papilla	Not to brush 48 h post-treatment, after 48 h, tooth brushing was advised in anterior aesthetic zone on the surgical site using soft bristle toothbrush
Mandel et al., 2020 [64]	98 injected (Revident HA:48, Flex Barrier HA:50) 62 negative control (Revident control:32, Flex Barrier control:30)	Both	Flex Barrier gel (Naturelize GmbH and Bio Science GmbH, Ransbach- Baumbach, Germany), two-thirds cross-linked and one-third non-cross-linked HA, Revident CLS LLC (Moscow, Russia), 1% HA formulation	Single injection	Non-surgical periodontal therapy 3–6 months prior	Three Step Technique (TST): (1) injection of the gel with a 30G needle along the mucogingival junction at the base of the papilla at 4–5 sites, creating depots of 0.1 ml per site, (2) injection of the gel into the attached gingiva at the base of the papilla at 2–3 sites, creating depots of 0.1 ml per site, (3) injection of the gel into the papilla 2–3 mm from its tip at one site, creating a depot of 0.1 ml	NR
Ni et al., 2019 [56]	22	Both	0.05–0.1 ml of 16 mg/ml HA gel (Qi sheng biological agent company Limited, Shanghai, China)	Multiple injections, repeated at 3 weeks, 6 weeks	When necessary, ultrasonic supragingival scaling to control the inflammation of the gingiva	The base of the deficient papillae	NR
Ni et al., 2021 [65]	62 (HA:31, Saline:31)	Max	0.05– 0.1 mL of 16 mg/ml HA gel (Qi sheng Biological Agent Company Limited, Shanghai, China)	Multiple injections, repeated at 3 weeks, 6 weeks	Initial periodontal therapy, scaling, root debridement, oral hygiene instructions	The base of the deficient papillae	NR
Patil et al., 2020 [58]	14	Max	< 0.2 mL commercially available and Food and Drug Administration approved HA gel	Multiple injections, repeated if needed after 3 weeks up to 2–3 times	NR	2–3 mm apical to the tip of the papilla	NR
Kapoor and Bhardwaj, 2022 [67]	60 (HA:30, Saline:30)	Max	0.2 ml of commercially available 2% HA gel	Multiple injections, repeated at 3 weeks and 3 months	Phase I periodontal therapy, patients education and motivation to maintain oral hygiene	Papilla was injected, then massaged in the coronal direction for around 1 min	Not to brush on the day of injection, to resume it after 24 h, not to floss in the treated area
Fakher et al., 2023 [68]	18 (HA:6, PRF:6, HA/PRF:6)	NR	0.2 ml of Cross-linked HA gel (MonaLisa, Genoss©. Co. South Korea)	Multiple injections, repeated at 3 weeks and 6 weeks	NR	In the papillae	NR
Lee et al., 2016 [59]	43	Max	0.01 cc of commercially available injectable HA gel (Teosyal Puresense Global ActionVR , Teoxane, Geneva, Switzerland)	Multiple injections, repeated at 3 weeks and 6 weeks	NR	45° angle, 2–3 mm below the interdental papilla tip, the interdental papilla tip was lightly massaged in the direction of the incisal edge with gauze	NR
Lee et al., 2016 [57]	57	Max	0.01 cc of HA gel (Teosyal Puresense Global Action®, Teoxane, Geneva, Switzerland)	Multiple injections, repeated up to 5 times at 3 weeks intervals	NR	45° angle, 2–3 mm apical to the involved papilla, then massaged in the incisal direction using gauze	NR
Singh and Vandana, 2019 [55]	35 (1% HA:16, 2% HA:7, 5% HA:12)	Both	< 0.2 ml (powder HA). 1%, 2%, and 5% of HA solutions were prepared by dissolving 10, 20, and 50 mg/ml of HA powder (Herb supply, LLC, LasVegas, NV)	Multiple injections, repeated at 2 weeks and 3 weeks	NR	2–3 mm apical to the coronal tip of the papilla, the area was gently massaged for 1 min	Avoid the use of dental floss at the treatment sites, the use of soft toothbrush coronal to the gingival margin
Sadat Mansouri et al., 2013 [40]	21	Max	< 0.2 ml	Multiple injections, repeated at 3 weeks and 3 months if needed	Phase 1 periodontal therapy if needed	2–3 mm apical to the coronal tip of the papilla	Not to brush their teeth at the day of injection and resume oral hygiene the day after using a soft toothbrush at the anterior teeth, place it coronal to gingival margin Not to use dental floss at the treatment sites
Shawky and Darwish, 2017 [69]	40 (HA:20, Calcium hydroxyapatite, Radiesse:20)	NR	< 0.2 ml of commercially available and FDA-approved HYALGAN® (HA gel)	Single injection	NR	2–3 mm apical to the coronal tip of the involved papillae	Not to brush their teeth at the day of injection and to resume oral hygiene measures on the following day by a soft toothbrush using Charter’s technique at the anterior teeth, not to use dental floss at the treatment sites for 4–6 weeks, 0.12% chlorhexidine mouthwash
Abdelkader and Ebrahem, 2022 [66]	42 (HA: 21, HA+ chitosan: 21)	NR	0.2 ml HA	Single injection	Full mouth mechanical debridement, supra and subgingival scaling and root planing with universal curette and ultrasonic instrument in two sessions, oral hygiene instructions, adjunctive chemical plaque control (Chlorhexidine mouthwash) twice daily for 1 week.	2–3 mm apical to the tip of the interdental papilla and directed coronally with an angulation of 45° to the long axis of the tooth and the bevel directed apically, the papilla was lightly massaged in an incisal direction for 1 min using gauze	NR
Firkova 2020 [50]	57	Max	HA gel, composed of a mixture of cross-linked (1.6%) and natural (0.2%), marketed as HyaDent BG (BioScience, Germany). The volume was tailored individually, till whitening of the adjacent tissue was noticed	Twice, repeated after 20 days	Scaling and root planing 4 weeks before injections	2–3 mm apical to the papilla tip directed coronally, few droplets of HyaDent BG were topically applied and massages onto the treated area	Not to brush for 24 h, after 24 h, start the routine oral hygiene measures, avoid dental floss at the treatment sites
Ebrahimi et al., 2023 [60]	20	NR	0.2 mL of HA gel (Syno- vial Forte 1.6% 32 mg, IBSA FARMACEUTICI, Italy, 2020)	Multiple injections, repeated three times at 2-week intervals	NR	Needle perpendicular to the injection sites with a 31G-0.3 ml insulin syringe, each papilla had two injection sites, one located 2–3 mm below the tip of the papilla and the other on the inter- dental tip, the injection was continued until the papilla became ischemic, then massaged towards the incisal edge to make the enlargement of the papilla just like a real embrasure	No brushing or flossing the region of injection, allowed to brush the coronal area of the gingiva the next day, but flossing was not permitted until 2 weeks after the last injection

HA: Hyaluronic acid; PRGF: Plasma rich growth factors; PRF: Platelet-rich Fibrin; NR: Not reported; Max: Maxilla; Mand, Mandible; h: Hours.

### Effect of hyaluronic acid injection on interdental papillae reconstruction

Digital photographs were employed by most of the included studies to assess the outcomes after hyaluronic acid injection; these outcomes included BTH, BTA, IDPH, PPI, BTW, CIPR, PIPR, HGP, IDPDS, PR, DP, CP-GM, IPW, PGO, and PT-CP with final follow up time ranging from 1 month to 2 years. Further details are presented in Table 5.

**Table 5:** Details of the reported outcomes in the included studies.

Author, Year	Papilla measurement tool	Follow up intervals	Outcomes measured (unit)	Outcomes details	Final conclusion
Turgut Çankaya and Tamam, 2020 [48]	Digital impressions	Baseline, 3 months, 1 year, 2 years	BTA (mm^2^) BTA (%)	**BTA:** Max: Baseline: 0.31 ± 0.05, 3 m: 0.14 ± 0.03, 1 y: 0.09 ± 0.02, 2 y: 0.07 ± 0.02, Mand, Baseline: 0.25 ± 0.05, 3 m: 0.11 ± 0.02, 1 y: 0.07 ± 0.01, 2 y: 0.06 ± 0.01 **BTA:** Max. 3 m: 54.21 ± 6.75, 1 y: 73.22 ± 5.60, 2 y: 79.35 ± 5.86, M and. 3 m: 57.24 ± 6.26, 1 y: 71.40 ± 3.32, 2 y: 78.71 ± 4.04	Repeated HA injections gradually increased papillary volume which was maintained for 2 years
Abdelraouf et al., 2019 [61]	Clinical measurement using periodontal probe & fabricated customised stent, digital clinical photographs	Baseline, 3 months, 6 months	(PT- CP) (mm) BTA (%)	**(PT- CP)**: HA: Baseline-3 m: -0.31 ± 0.25, 3–6 m: -0.06 ± 0.17, Baseline-6 m: -0.25 ± 0.26, Saline: Baseline-3 m: -0.07 ± 0.18, 3–6 m: 0.04 ± 0.13, Baseline-6 m: -0.03 ± 0.13 **BTA:** HA: Baseline-3 m: -36.5 ± 24.4, 3–6 m: -11.8 ± 30.3, Baseline-6 m: -45.0 ± 28.5, Saline: Baseline-3 m: -0.9 ± 10.6, 3–6 m: 0.9 ± 8.9, Baseline-6 m: -2.0 ± 11.4	The use of commercially available HA gel for the treatment of interdental papillary deficiency was effective with promising levels of patients’ satisfaction
Bertl et al., 2017 [62]	Clinical measurements, digital clinical photograph	Baseline, 3 months, 6 months	PT-CP (mm) BTA (mm^2^)	**PT-CP**: Saline: Baseline: 2.3 ± 1.2, 3 m: 2.2 ± 1.2, 6 m: 2.2 ± 1.2, HA: Baseline: 2.0 ± 1.1, 3 m: 1.8 ± 0.7, 6 m: 1.9 ± 0.8 **BTA:** Saline: Baseline: 0.51 ± 0.25, 3 m: 0.49 ± 0.23, 6 m: 0.54 ± 0.27, HA: Baseline: 0.51 ± 0.31, 3 m: 0.47 ± 0.20, 6 m: 0.52 ± 0.26	Solely injecting this specific HA gel with this particular 3-step injection technique to augment deficient interproximal papillae at implant-supported crowns in the anterior maxilla does not have any appreciable clinical effect
Bal et al., 2023 [63]	Clinical measurement, digital clinical photographs	Baseline, 3 weeks, 6 weeks, 12 weeks	BTW(mm) BTH (mm)	**BTW:** HA: Baseline: 0.94 ± 0.35, 3 w: 0.81 ± 0.29, 6 w: 0.68 ± 0.32, 12 w: 0.60 ± 0.36, HA+PRGF: Baseline: 0.77 ± 0.24, 3 w: 0.69 ± 0.21, 6 w: 0.64 ± 0.21, 12 w: 0.54 ± 0.19 **BTH:** HA: Baseline: 1.28 ± 0.69, 3 w: 1.18 ± 0.68, 6 w: 1.04 ± 0.53, 12 w: 0.89 ± 0.61, HA+PRGF: Baseline: 1.12 ± 0.32, 3 w: 0.90 ± 0.11, 6 w: 0.56 ± 0.20, 12 w: 0.20 ± 0.21	HA gel is a promising injectable agent in the minimally invasive treatment of interdental papillary deficiency, the addition of PRGF holds promise for further investigations
Awartani and Takis, 2016 [49]	Digital clinical photographs	Baseline, 4 months, 6 months	BTA (mm^2^)	**BTA:** Baseline: 1.24 ± 1.84, 4 m: 0.57 ± 0.93, 6 m: 0.71 ± 0.74	The use of a commercially available HA gel for the treatment of aesthetic interdental papilla deficiency was somewhat effective, when assessed up to 6 months with promising levels of patient satisfaction, future studies are needed to ascertain long-term outcomes and determine the appropriate time period for re-treatment, identify pre-treatment determinants of positive outcomes and patient satisfaction, and perform comparisons between different available materials
da Silva et al., 2023 [51]	Standardised photographic and CAD/CAM analysis	Baseline, 1 month, 2 months, 3 months, 4 months	PGO (mm) PR (%)	**PGO** (photographs): 1 m: 0.08 ± 0.21, 2 m: 0.04 ± 0.16, 3 m: 0.08 ± 0.3, 4 m: 0.22 ± 0.29 (CAD/CAM): 1 m: 0.13 ± 0.08, 2 m: 0.24 ± 0.14, 3 m: 0.41 ± 0.21, 4 m: 0.38 ± 0.21 **PR:** (CAD/CAM): 1 m: 30.41 ± 23.4, 2 m: 39 ± 26.1, 3 m: 58 ± 32.9, 4 m: 49.1 ± 46.1	The application of injectable HA was effective in filling papillary tissue in the aesthetic area
Alhabashneh et al., 2021 [52]	Digital clinical photographs	Baseline, 3 weeks, 3 months, 6 months	BTH (mm)	**BTH:** Baseline: 2.14, 3 w: 1.97, 3 m: 1.31, 6 m: 1.52	The use of commercially available HA gel for the treatment of black triangles due to interdental papillae loss is a procedure with promising results over the first 6 months after injection, future trials are needed to determine the most appropriate protocol of HA injection and to identify the pretreatment determinants for better clinical outcomes
Pitale et al., 2021 [53]	Standardised clinical digital photographs	Baseline, 3 months, 6 months	CP-GM (mm) BTH (mm) IPW (mm) BTW (mm)	**CP-GM:** Baseline: 1.72 ± 1.17, 3 M: 0.56 ± 0.86, 6 m: 0.64 ± 0.95 **BTH**: Baseline: 1.69 ± 1.25, 3 m: 0.58 ± 0.90, 6 m: 0.63 ± 0.92 **IPW**: Baseline: 1.28 ± 0.54, 3 m: 0.40 ± 0.57, 6 m: 0.44 ± 0.58 **BTW**: Baseline: 1.24 ± 0.69, 3 m: 0.38 ± 0.58, 6m: 0.42 ± 0.60	Injectable HA gel is a promising minimally invasive therapy for enhancing papillary aesthetics, the reconstruction was achieved and maintained for 6 months, long-term follow-up with more sample size will further establish HA as a minimally invasive technique
Becker et al., 2010 [39]	Digital clinical photographs	Not standardised, patients were followed up from 6 to 25 months	ΔDP (%)	**ΔDP:** 91.1 ± 12	Small papillary deficiencies between implants and teeth can be enhanced by minimally invasive injection of a hyaluronic gel, improvements were maintained for a range of 6–25 months
Patel et al., 2017 [54]	Digital clinical photographs	Baseline, 1 month, 3 month, 6 month, up to 9 months	CIPR (%) PIPR (%) PR (%)	**CIPR:** 62.5 **PIPR:** 37.5 **PR:** 1 m: 1–15, 3 m: 12–83, 6 m: 22–100	Class I and class II gingival black triangles can be enhanced by injecting HA gel, injectable HA gel may be a promising treatment for enhancing gingival papillary aesthetics
Mandel et al., 2020 [64]	Digital clinical photographs	Baseline, immediately after injection, 1 week, 1 month	IDPDS (%)	**IDPDS:** HA Revident: Baseline:100 ± 0, Immediately after treatment: 81.0 ± 16.38, 1 w: 84.8 ± 18.9, 1 m: 86.1 ± 22.6 Revident negative control: Baseline: 100 ± 0, Immediately after treatment: 100 ± 0, 1 w: 99.3 ± 3.6, 1 m: 100.4 ± 4.6 Flex Barrier HA: Baseline: 100 ± 0, Immediately after treatment: 83.8 ± 17.1, 1 w: 91.2 ± 13.24, 1 m: 96.0 ± 8.8 Flex Barrier negative control: Baseline: 100 ± 0, Immediately after treatment: 100 ± 0, 1 w: 100.0 ± 1.1, 1 m: 100.1 ± 0.9	HA injection is applicable for interdental papilla defect treatment, single-injection protocol Revident showed longer lasting effects than Flex Barrier, future need for well-designed randomised clinical trials to determine the optimal arrangements for treating gingival ‘black triangles’ with multiple injections of HA
Ni et al., 2019 [56]	Standardised digital clinical photographs	Baseline, 3 months, 6 months, 12 months	HGP (mm) BTA (mm^2^)	**HGP:** Baseline: 2.90 ± 1.30, 3 m: 3.21 ± 1.20, 6 m: 3.35 ± 1.11, 1 y: 3.30 ± 1.11 **BTA:** Baseline: 1.355 ± 0.779, 3 m: 1.043 ± 0.748, 6 m: 0.994 ± 0.739, 1 y: 1 ± 0.831	Injection of HA gel has an appreciable effect on the augmentation of deficient gingival papilla in the natural teeth, future research efforts should focus on the identification of gingival papilla-deficient patient subgroups that demonstrate a more effective response to HA injection, determination of the injection method and cycle, further elucidation of the mechanisms that induce the gingival papilla to grow and the potential for combination therapy
Ni et al., 2021 [65]	Standardised digital clinical photograph	Baseline, 6 months, 12 months	HGP (mm) BTA (mm^2^)	**HGP:** HA: Baseline: 3.25 ± 1.30, 6 m: 3.45 ± 1.27, 1 y: 3.53 ± 1.25, Saline: Baseline: 2.99 ± 1.42, 6 m: 3.12 ± 1.36, 1 y: 3.26 ± 1.39 **BTA:** HA: Baseline: 1.90 ± 1.37, 6 m: 1.65 ± 1.32, 1 y: 1.45 ± 1.16, Saline: Baseline: 1.78 ± 1.31, 6 m: 1.63 ± 1.26, 1 y: 1.46 ± 1.26	The injection of HA could increase the gingival papilla height for gingival papilla defects and reduce the area of the resulting black triangles, no statistically significant improvement comparing with physiological saline, long-term multicentre clinical study should be carried out to standardize the protocol for HA injection, optimise the injection concentration and interval, and identify the determinants affecting the clinical outcomes
Patil et al., 2020 [58]	Clinical digital photographs	Baseline, 3 months	ΔBTH (mm) ΔBTW (mm) ΔBTA (mm^2^) CIPR (%) PIPR (%)	**ΔBTH:** 3 m: 0.85 ± 0.28 **ΔBTW:** 3 m: 0.34 ± 0.15 **ΔBTA:** 3 m: 0.25 ± 0.12 **CIPR:** 3 m: 57.14 **PIPR:** 3 m: 42.85	The application of HA gel was somehow effective for interdental papilla reconstruction and might be used as a minimally invasive technique for the reconstruction of interdental papilla
Kapoor and Bhardwaj, 2022 [67]	Standardised digital clinical photographs	Baseline, 3 weeks, 3 months, 6 months	BTW (mm) BTH (mm) BTA (mm^2^)	**BTW:** HA: Baseline: 2.81 ± 1.14, 3 w: 2.10 ± 1.06, 3 m: 1.21 ± 0.64, 6 m: 0.55 ± 0.46, Saline: Baseline: 2.96 ± 1.00, 3 w: 3.17 ± 0.97, 3 m: 2.96 ± 0.99, 6 m: 2.93 ± 0.97 **BTH:** HA: Baseline: 3.86 ± 2.01, 3 w: 2.80 ± 1.59, 3 m: 1.32 ± 0.76, 6 m: 0.58 ± 0.40, Saline: Baseline: 5.02 ± 1.84, 3 w: 5.31 ± 1.44, 3 m: 4.97 ± 1.45, 6 m: 4.95 ± 1.34 **BTA:** HA: Baseline: 1.58 ± 0.46, 3 w: 1.20 ± 0.37, 3 m: 0.98 ± 0.15, 6 m: 0.42 ± 0.26, Saline: Baseline: 1.54 ± 0.54, 3 w: 1.86 ± 0.62, 3 m: 1.93 ± 0.55, 6 m: 1.85 ± 0.52	HA is a non-invasive voluminising alternative treatment modality for interdental papilla deficiency, it is biocompatible, as it does not cause an adverse reaction
Fakher et al., 2023 [68]	Clinical digital images	Baseline, 3 weeks, 6 weeks, 3 months, 6 months	ΔBTH (%)	**ΔBTH**: HA: 3 w: 17.65%, 6 w: 31.18%, 3 m: 57.65%, 6 m: 92.35%, iPRF: 3 w: 15.15%, 6: 30.30%, 3 m: 39.39%, 6 m: 53.54%, HA/iPRF: 3 w: 12.00%, 6 w: 22.80%, 3 m: 34.80%, 6 m: 46.80% **BTH**: HA: Baseline: 1.73, 3 w: 1.4, 6 w: 1.17, 3 m: 0.72, 6 m: 0.13, iPRF: Baseline: 1.98, 3 w: 1.68, 6 w: 1.38, 3 m: 1.2, 6 m: 0.92, HA/iPRF: Baseline: 2.5, 3 w: 2.2, 6 w: 1.93, 3 m: 1.63, 6 m: 1.33	HA and iPRF injections can be effective non-invasive treatment method for gingival black triangles
Lee et al., 2016 [59]	Standardised digital clinical photographs	Baseline, 6 months	BTA (mm^2^) BTH (mm) BTW (mm) CIPR (%) PIPR (%)	**BTH:** Baseline: 0.93, 6 m: 0.71 ± 0.27, **BTW:** Baseline: 0.41 ± 0.18, 6 m: 0.32 ± 0.13 **BTA:** Baseline: 0.24 ± 0.19, 6 m: 0.20 ± 0.13 **CIPR:** 67.44 **PIPR:** 32.56	Interdental papilla reconstruction using an injectable HA gel can be a viable treatment option for interdental papilla deficiencies in small areas
Lee et al., 2016 [57]	Standardised clinical photographs	Baseline, 6 months	ΔBTH (mm) ΔBTW (mm) ΔBTA (mm^2^) CIPR (%) PIPR (%)	**ΔBTH:** 0.70 ± 0.29 **ΔBTW:** 0.30 ± 0.13 **ΔBTA:** 0.21 ± 0.14 **CIPR**: 63.16 **PIPR**: 36.84	Interdental papilla reconstruction using injectable HA gel allowed successful reconstruction when the CP-BC ≤ 6 mm, but for the CP-BC > 6 mm, the increased distance resulted in a decreased IPRR, the CP-BC is closely associated with the efficacy of HA gel injection for the reconstruction of deficient interdental papillae in the upper anterior area
Singh and Vandana, 2019 [55]	Clinical photographs, occlusal stent and cast model	Baseline, 6 months	BTA (mm)	**BTA:** *Cast model:* HA 1%: Baseline: 3.59 ± 0.93, 1 m: 3.09 ± 1.15, 3 m: 3.21 ± 0.79, 6 m: 3.43 ± 0.85, HA 2%: Baseline: 3.85 ± 1.4, 1 m: 3.64 ± 0.85, 3 m: 3.64 ± 0.8, 6 m: 4.14 ± 1.2, HA 5%: Baseline: 3.91 ± 1.81, 1 m: 3.16 ± 1.68, 3 m: 3.1 ± 1.6, 6 m: 3.2 ± 1.6, *Photographs:* HA 1%: Baseline: 474.3 ± 219.9, 1 m: 393.7 ± 2.5, 3 m: 385.3 ± 212.1, 6 m: 407.1 ± 299.1, HA 2%: Baseline: 324.1 ± 287.6, 1 m: 292.7 ± 256.7, 3 m: 304.5 ± 265.4, 6 m: 318.8 ± 266.9, HA 5%: Baseline: 333.5 ± 210.4, 1 m: 195.2 ± 194.4, 3 m: 190.7 ± 193.3, 6 m: 201 ± 208.9	HA injection is cost-effective and safe to use in treatment of deficient papilla with 5% of HA had minimal rebound, long-term studies would throw more insight, further histologic studies are required to assess the mechanism of hyaluronic action
Sadat Mansouri et al., 2013 [40]	Digital clinical photographs	Baseline, 3 weeks, 3 months, 6 months	BTA (Pixels %)	**BTA:** 3 w: 3/38 ± 3/07, 3 m: 29/52 ± 18/72, 6 m: 47/33 ± 20/20	Application of HA gel was somehow effective for interdental papilla reconstruction and may be used as a non-invasive technique for reconstruction of interdental papilla
Shawky and Darwish, 2017 [69]	Clinical digital photographs	Baseline, 3 weeks, 3 months, 6 months	IDPH (mm) PPI (score)	**IDPH:** HA: Baseline: 3.81 ± 0.49, 3 w: 4.31 ± 0.67, 3 m: 4.28 ± 0.64, 6 m: 4.10 ± 0.59, Radiesse: Baseline: 3.81 ± 0.49, 3 w: 5.12 ± 0.40, 3 m: 5.09 ± 0.43, 6 m: 5.10 ± 0.43 **PPI:** HA: Baseline: Score 1: 0%, Score 2: 85%, Score 3: 15%, 3 w: Score 1: 75%, Score 2: 25%, Score 3: 0%, 3 m: Score 1: 55%, Score 2: 45%, Score 3: 0%, 6 m: Score 1: 50%, Score 2: 45, Score 3: 5%, Radiesse: Baseline: Score 1: 0%, Score 2: 80%, Score 3: 20%, 3 w: Score 1: 90%, Score 2: 10%, Score 3: 0%, 3 m: Score 1: 90%, Score 2: 10%, Score 3: 0%, 6 m: Score 1: 85%, Score 2: 15%, Score 3: 0% **Mean PPI:** HA: Baseline: 2.15 ± 0.37, 3 w: 1.25 ± 0.44, 3 m: 1.45 ± 0.51, 6 m: 1.55 ± 0.60, Radiesse: Baseline: 2.20 ± 0.41, 3 w: 1.10 ± 0.31, 3 m: 1.10 ± 0.31, 6 m: 1.15 ± 0.37	Both HA and Radiesse were effective and safe for interdental papilla augmentation, though, Radiesse gel offered more superior improvement concerning its immediate volumising as well as its long-lasting effect
Abdelkader and Ebrahem, 2022 [66]	Clinical assessment	Baseline, 3 months, 6 months	BTH (mm) BTA (mm^2^)	**BTH:** HAC: Baseline: 1.98 ± 0.45, 3 m: 1.71 ± 0.45, 6 m: 1.33 ± 0.47, HA: Baseline: 2.23 ± 0.40, 3 m: 2.03 ± 0.40, 6 m: 1.79 ± 0.34 **BTA**: HAC: Baseline: 0.66 ± 0.20, 3 m: 0.34 ± 0.11, 6 m: 0.19 ± 0.07, HA: Baseline: 0.68 ± 0.15, 3 m: 0.42 ± 0.08, 6 m: 0.24 ± 0.05	Treatment of black triangle of interdental papilla using injectable HA gel alone or carboxymethyl chitosan hydrogel and HA gel show improvement in clinical outcomes, although the reduction of the black triangles after 6 months in HAC was more but not statistically significant compared to HA alone
Firkova 2020 [50]	Clinical photographs	Baseline, 1 month, 3 months, 6 months	BTH (mm), CIPR (%) PIPR (%)	**BTH:** Max. Baseline: 0.31 ± 0.05, 3 m: 0.14 ± 0.03, 1 y: 0.09 ± 0.02, 2 y: 0.07 ± 0.02 – Mand. Baseline: 0.25 ± 0.05, 3 m: 0.11 ± 0.02, 1 y: 0.07 ± 0.01, 2 y: 0.06 ± 0.01 **CIPR**: 32.7 **PIPR:** 67.3	Minimally invasive reconstruction of lost papillae with HA is an easy, affordable and predictable technique, which satisfies the aesthetic demands of both patients and dentists for a period of at least 6 months, minimally invasive management of deficient papillae with HA responds to the growing public demands for aesthetics and harmonious smile of patients
Ebrahimi et al., 2023 [60]	Standard clinical photographs	Baseline, 2 weeks, 4 weeks, 3 months, 6 months	BTA (mm^2^) IDPH (mm) PT-CP (mm)	**BTA:** Baseline: 2.09 ± 1.22, 2 w: 1.17 ± 0.60, 4 w: 0.81 ± 0.50, 3 m: 0.54 ± 0.45, 6 m: 0.38 ± 0.41 **IDPH:** Baseline: 2.52 ± 0.82, 2 w: 3.20 ± 0.75, 4 w: 3.53 ± 0.89, 3 m: 3.65 ± 1.01, 6 m: 3.96 ± 0.84 **PT-CP:** Baseline: 1.94 ± 0.65, 2 w: 1.29 ± 0.50, 4 w: 0.98 ± 0.54, 3 m: 0.57 ± 0.33, 6 m: 0.36 ± 0.29	Injection of 1.6% viscosity HA gel at two points of the interdental papilla was effective in reconstructing the interdental papilla at the aesthetic zone in 6 months follow ups

BTH: Black triangle height; BTA: Black triangle area; IDPH: Interdental papilla height; PPI: Papillary presence index; BTW: Black triangle width; CIPR: Complete interdental papilla reconstruction; PIPR: Partial interdental papilla reconstruction; HGP: Height of gingival papilla; IDPDS: Interdental papillary defect size; PR: Papilla reconstruction; DP: Deficient papilla; CP-GM: Contact point to gingival margin distance; IPW: Interproximal width; PGO: Papilla gain oscillation; PT-CP: Papilla tip to contact point; PRGF: Plasma rich growth factors; iPRF: injectable platelet rich fibrin; HA: Hyaluronic acid; HAC: Hyaluronic acid + chitosan; CP-PC: Contact point to bone crest; IPRR: Interdental papilla reconstruction rate; Max, Maxilla; Mand: Mandible; CAD/CAM: Computer-aided design and computer-aided manufacturing; W: Weeks; M: Months; Y: Years.

All the included studies revealed positive results following hyaluronic acid injection as a minimally invasive method to reconstruct deficient interdental papilla, particularly in patients with thick gingival biotype [56]. However, only one study stated that there was no considerable clinical outcome obtained after injecting deficient papilla at implant-supported crowns in the anterior maxilla [62]. Despite the use of different commercial brands of hyaluronic acid materials, the included studies verified the clinical efficiency of all these different types, as all were associated with similar positive results. Yet, some had longer lasting results; Revident HA had longer lasting results than Flex Barrier HA [64]. Moreover, it was found that the higher the concentration of hyaluronic acid, the more favourable the outcome; the use of 5% of hyaluronic acid material has yielded more significant improvement in comparison to 1% and 2% concentrations [55]. In addition, reconstruction of lost interdental papilla was attained with the injection of hyaluronic acid alone as well in combination with other material; platelet-rich fibrin [68], plasma rich growth factor [63], and carboxymethyl chitosan [66].

### Patients’ satisfaction and reported complications

Three studies evaluated patients’ satisfaction utilising the visual analogue scale (VAS) [61, 66, 68] while two utilised self-reported questionnaires [49, 55] in which all reported an increase in satisfaction following hyaluronic acid injection for reconstruction of lost interdental papilla. Other two studies employed VAS for pain assessment following injection. In one study, the reported pain was less in the hyaluronic acid group in comparison to the saline group [62] while the other study reported marked reduction in the pain level at the third day after injection with no pain reported after the seventh day [52]. Complications other than pain were bleeding which was managed by pressure application [56], limited swelling and tenderness at the injection site which lasted for 3 days [49], and swelling of the lip with painless granuloma in the gingiva above the mucogingival junction which lasted for more than 4 weeks but was not detectable after 3 months [62].

### Quality assessment and risk of bias in the included studies

**Figure 2:**
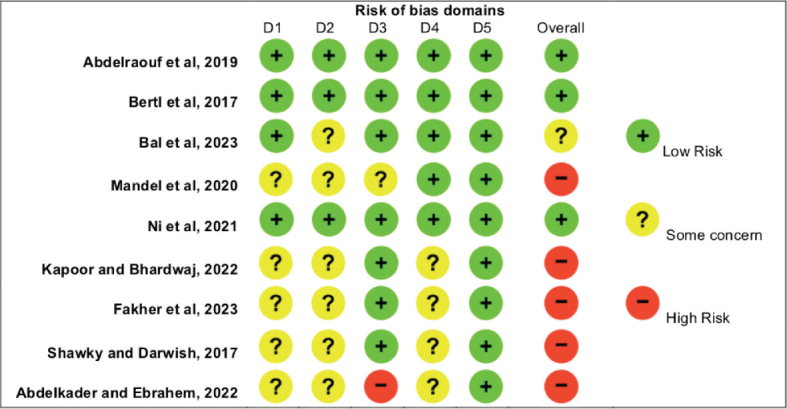
Risk of bias assessment using the RoB2: the revised Cochrane risk of bias tool for randomised clinical trials.

The Newcastle Ottawa quality assessment tool revealed generally good quality among the included prospective cohort studies [40, 48, 49–54, 56–60] except for two with poor quality according to the scale [39, 55]. Detailed information regarding the three domains is presented in Table 6. The RoB2: the revised Cochrane risk of bias tool for randomised clinical trials revealed three studies with low risk [61, 62, 65], one with some concern [63], and five studies with high risk [64, 66–69]. Detailed information regarding the five domains is presented in Figure 2.

**Table 6:** Quality assessment of prospective clinical cohort studies using the Newcastle-Ottawa scale.

Study	Newcastle-Ottawa scale			
	Selection	Comparability	Outcome	Overall
Turgut Çankaya and Tamam, 2020 [48]	***	*	***	Good
Awartani and Takis, 2016 [49]	***	*	***	Good
da Silva et al., 2023 [51]	***	*	**	Good
Alhabashneh et al., 2021 [52]	***	*	***	Good
Pitale et al., 2021 [53]	***	*	***	Good
Becker et al., 2010 [39]	***	**-**	***	Poor
Patel et al., 2017 [54]	***	*	***	Good
Ni et al., 2019 [56]	***	*	***	Good
Patil et al., 2020 [58]	***	*	**	Good
Lee et al., 2016 [59]	***	*	***	Good
Lee et al., 2016 [57]	***	*	***	Good
Singh and Vandana, 2019 [55]	***	**-**	***	Poor
Sadat Mansouri et al., 2013 [40]	***	*	***	Good
Firkova 2020 [50]	***	*	***	Good
Ebrahimi et al., 2023 [60]	***	*	***	Good

## Discussion

This systematic review aimed to analyse the available evidence on the effect of hyaluronic acid injection in reconstructing lost interdental papilla. In 2010, Becker et al. first proposed this innovative technique in a pilot study [39]. Hyaluronic acid is a major element of the extracellular matrix in the gingival tissue, which promotes the gingival fibroblasts cells capacity for migration and proliferation [32, 33]. This was confirmed in two clinical studies, which additionally took into account an in vitro assessment of human gingival fibroblasts cells migration and proliferation [65, 67]. This could possibly justify the use of hyaluronic acid injection in the reduction of black triangle and the reconstruction of lost interdental papilla.

Based on the clinical studies included in this systematic review of the literature, hyaluronic acid injection showed positive result and contributed effectively to the reconstruction of lost interdental papilla in natural anterior teeth with partial to CIPR at the deficient sites [39, 40, 48–61, 63–69]. Only one study reported that this minimal change in the papilla gain might not be clinically appreciable; however, this could be attributed to the fact that this clinical study was performed only on papilla in implants and not natural teeth [62]. The histological characteristics around natural teeth are different compared to peri-implant tissue, mainly, the absence of periodontal ligament around implants resulting in reduced vascularity and fewer fibroblasts [72], thus, restrict hyaluronic acid’s rheological impact at the injected site and limit the outcome at peri-implant tissue.

In addition to these positive reported results, hyaluronic acid injection seems to be a preferred treatment option among patients, not just due to its non-invasive nature but also because of its cost-effective outcome. Other non-invasive treatment modalities, which include orthodontic treatment [24] and restorative treatment [25], are more expensive and time-consuming and could last for months, if not years, depending on the severity of the case, compared to hyaluronic acid injection, in which the result can seem instantly right after the injection procedure. Hyaluronic acid injections appear to outperform the other non-invasive techniques economically, despite the possible need for follow-up injections.

The outcome of hyaluronic acid injection can be influenced by several variables. The viscosity and concentration of the injected hyaluronic acid can relate to the flexibility of the material. A low viscosity material was found to be compatible for filling surface areas while in deep areas high viscosity exhibited superior outcomes [73]. Most of the studies included in this systematic review utilised average viscosity and concentration of 16–25 mg/ml, medium to high viscosity, in which all were effective, thus, hyaluronic acid injection was found to be effective in papillary reconstruction regardless of the viscosity of the material. Singh and Vandana compared different hyaluronic acid concentrations, 1%, 2%, and 5%. They reported that 5% showed better outcome compared to 2% and 1% [55]. However, this was the only study that used hyaluronic acid in the form of powder diluted in a solution in comparison to all the other studies, which employed hyaluronic acid in the form of gel. Hence, it is crucial to interpret this result cautiously with more clinical trials need to determine the adequate concentration associated with the most stable results in the long-term. In addition, the injected arch was found to be a considerable variable by Alhabashneh et al. in which better papillary fill was reported in the maxilla compared to the mandible [52]. The authors justified this finding due to the presence of more keratinized gingiva and symmetric teeth form in the maxillary arch [52]. Ni et al. reported that thick gingival phenotype was associated with relapse in the papillary gain after 1 year of hyaluronic acid injection resulting in recurrence of the interdental black triangles, although this phenotype was associated with better initial outcomes after the injection [56]. This could be related to the thick underlying connective tissue, subsequently; more abundant gingival fibroblasts and collagen fibres present [74]. However, more studies are necessary for further investigation of the influence of this variable. Another variable recognised by Lee et al. is the degree of the papillary loss. It was reported that large black triangles are less predicted and do not respond as positively as the small deficiencies following the injection of hyaluronic acid [59]. Most of the included studies in this review were associated with limited interdental papillary loss, class I and II according to Nordland and Tarnow classification [49, 50, 52–54, 56, 58, 60, 61, 63–68], score 2 according to the modified papilla index score [48, 62], and score 2 and 3 according to Cardaropoli classification [55] in which all agreed on favourable outcomes of this minimally invasive technique. Therefore, it can be stated that hyaluronic acid injection should be considered in the reconstruction of lost interdental papillae that do not involve significant amounts of papillary tissue loss and large BTA. When considering the long-term stability of the outcome, Becker et al. reported that completely reconstructed papilla was stable for about 2 years [39]. Similarly, Lee et al. revealed no relapse in completely reconstructed papilla, in contrast to partially reconstructed papilla, which was not stable over 6 months [59].

Alternative injected materials to hyaluronic acid were explored in several clinical trials. Bertl et al. injected saline solution for reconstruction of lost interdental papilla around implants and found no clinical difference in the papillary volume gain compared to hyaluronic acid [62]. In contrast, Abdelraouf et al. reported superior clinical results for interdental papilla reconstructed using hyaluronic acid injection compared to saline solution injection [61], which was due to the anatomical dissimilarity of the injected tissue, as Bertl et al. injected interdental papilla adjacent to implants while Abdelraouf et al. injected interdental papilla around natural teeth. Similar results reported by Ni et al. [65] and Kapoor and Bhardwaj [67], which was in agreement with Abdelraouf et al. [61]. Fakher et al. injected platelet rich fibrin for papillary reconstruction in comparison to hyaluronic acid and concluded that both materials are clinically effective alternatives [68]. Shawky et al. investigated the effect of calcium hydroxyapatite material (Radiesse), which is commonly used in cosmetic medicine, compared to hyaluronic acid. A significant clinical outcome was observed after injection in both materials with greater papillary reconstruction and longer-lasting effect related to calcium hydroxyapatite material [69]. However, additional clinical trials are essential to confirm this superior outcome.

The latest published systematic review on this topic by Faé et al. included 16 studies; however, their database search was limited, up to May 2022 [75]. The present study provided a broader and more robust review of the available literature with more articles included resulting in a total of 24 clinical studies. The significance of this study is to provide a detailed overview of hyaluronic acid injection for the treatment of lost interdental papilla in adult human, as there is no clear consensus on this topic. There is still uncertainty about the proper injection technique, the hyaluronic acid material to be used, the adequate amount of injected material, and the need for repeated injections to maintain the long-term effect of papilla reconstruction. Furthermore, adverse effects were reported; thus, it is challenging to preclude their occurrence. For these reasons, it is essential to investigate the current literature to generate a better understanding of this topic. A systematic review was imperative not only to comprehensively investigate the long-term effect of hyaluronic acid injection for reconstruction of lost interdental papilla but also to evaluate the safety of these injections and identify the potential factors that could lead to adverse effects. Such a review would provide valuable insight for dental practitioners, facilitating treatment decision-making and optimising patient outcomes. This was employed in this systematic review as it investigated both the effect of hyaluronic acid injection as well as the reported negative adverse complications associated with these injections, which is considered a strength of this systematic review. Another strength of this study is the use of manual search in addition to electronic databases. Also, there was no lower border or starting date for the search, and no studies were excluded due to a different language other than English; thus, hypothetically, we can state that there was no language restriction. Moreover, because of the limited available evidence and in order to systematically assess the studies on this topic, the authors did not confine this systematic review to randomised clinical trials only but included adult human studies of prospective nature; randomised clinical trials, and prospective cohort studies to provide a broader range of data to be included within this systematic review. However, no retrospective studies were included to avoid potential bias in the reported results.

Despite the reported favourable outcomes in this systematic review regarding the injection of hyaluronic acid for reconstruction of lost interdental papilla, some limitations should be noted. The major limitation is the high heterogeneity in these studies, which was attributed to the methodological diversity. These include, the use of different hyaluronic acid materials, the lack of standardised injection technique, different location of the injection, material’s concentration, frequency of injection, and dissimilar parameters measured to assess the outcome. Moreover, since the outcome of the papillary reconstruction was measured utilising images obtained from clinical photography or digital impressions, it is essential to emphasise the need for a standardised approach to ensure the reproducibility of these images at different time points. Some of the included studies implemented the needed standardisation; yet, the standardisation technique was not consistent making the comparison among these studies difficult. In addition, photographic analysis is a two-dimensional method, which might fail to capture the actual papillary reconstruction outcome and volumetric changes, as the interdental papilla is a three-dimensional structure in nature. This heterogeneity among the included studies made the quantitative analysis and meta-regression not feasible. Further limitations are the relatively small number of injected papillae in each clinical study and the short follow up time. Further well-conducted randomised clinical trials with large sample size and longer follow up period are essential in order to attain more precise results upon standardising the clinical methodology and outcome assessment procedure.

## Conclusion

Despite the high heterogeneity due to the absence of clear and standardised protocol, the use of hyaluronic acid injection seems to be an effective minimally invasive approach in treating black triangles and reconstructing lost interdental papilla in the short-term as the results pointed toward positive outcomes with minimal adverse reaction and promising levels of patients’ satisfaction. This could suggest the possible use of this material for other medical procedures. Further longer-term well-designed randomised clinical trials employing standardised procedures are essential to validate this treatment and provide better quality of evidence.

## Data Availability

No new data were created or analysed in this study. Data sharing is not applicable to this article.

## References

[1] LaVacca MI, Tarnow DP, Cisneros GJ. Interdental papilla length and the perception of aesthetics. Pract Proced Aesthet Dent. 2005;17:405–12. 16185029

[2] Hassell TM. Tissues and cells of the periodontium. Periodontol 2000. 1993;3:9–38. 10.1111/j.1600-0757.1993.tb00230.x 9673156

[3] Cohen B. Morphological factors in the pathogenesis of the periodontal disease. Br Dent J. 1959;107:31–9.

[4] Tarnow DP, Magner AW, Fletcher P. The effect of the distance from the contact point to the crest of bone on the presence or absence of the interproximal dental papilla. J Periodontol. 1992;63:995–6. 10.1902/jop.1992.63.12.995 1474471

[5] Bennani V, Ibrahim H, Al-Harthi L, Lyons KM. The periodontal restorative interface: esthetic considerations. Periodontol 2000. 2017;74:74–101. 10.1111/prd.12191 28429482

[6] Chow YC, Eber RM, Tsao YP, Shotwell JL, Wang HL. Factors associated with the appearance of gingival papillae. J Clin Periodontol. 2010;37:719–27. 10.1111/j.1600-051X.2010.01594.x 20618545

[7] Rotundo R, Bassarelli T, Pace E, Iachetti G, Mervelt J, Pini Prato G. Orthodontic treatment of periodontal defects. Part II: a systematic review on human and animal studies. Prog Orthod. 2011;12:45–52. 10.1016/j.pio.2011.02.008 21515231

[8] Addy M, Hunter ML. Can tooth brushing damage your health? Effects on oral and dental tissues. Int Dent J. 2003;53:177–86. 10.1111/j.1875-595x.2003.tb00768.x 12875306

[9] Alahmari F. Reconstruction of lost interdental papilla: a review of nonsurgical approaches. J Dent Med Sci. 2018;17:59–65.

[10] Nordland WP, Tarnow DP. A classification system for loss of papillary height. J Periodontol. 1998;69:1124–6. 10.1902/jop.1998.69.10.1124 9802711

[11] Cho HS, Jang HS, Kim DK, Park JC, Kim HJ, Choi SH, et al. The effects of interproximal distance between roots on the existence of interdental papillae according to the distance from the contact point to the alveolar crest. J Periodontol. 2006;77:1651–7. 10.1902/jop.2006.060023 17032106

[12] Hochman MN, Chu SJ, Tarnow DP. Maxillary anterior papilla display during smiling: a clinical study of the interdental smile line. Int J Periodontics Restorative Dent. 2012;32:375–83. 22577642

[13] Cunliffe J, Pretty I. Patients’ ranking of interdental ‘black triangles’ against other common aesthetic problems. Eur J Prosthodont Restor Dent. 2009;17:177–81. 20158060

[14] Muthukumar S, Ajit P, Sundararajan S, Rao SR. Reconstruction of interdental papilla using autogenous bone and connective tissue grafts. J Indian Soc Periodontol. 2016;20:464–7. 10.4103/0972-124X.193164 28298832 PMC5341325

[15] Yi Z, Miao X, Wang L, Zhang G, Wu Y. A customized subepithelial connective tissue graft for interdental papilla reconstruction and soft tissue augmentation. J Esthet Restor Dent. 2022;34:451–60. 10.1111/jerd.12858 34964233

[16] Henriques PG, Okajima LS, Siqueira S Jr. Surgical reconstruction of the interdental papilla: 2 case reports. Gen Dent. 2018;66:e1–4. 29964255

[17] Sharma E, Sharma A, Singh K. The role of subepithelial connective tissue graft for reconstruction of interdental papilla: clinical study. Singapore Dent J. 2017;38:27–38. 10.1016/j.sdj.2017.05.001 29229072

[18] Nordland WP. Restoration of lost interdental papilla: a surgical technique. Compend Contin Educ Dent. 2018;39:544–9. 30188151

[19] Zhang X, Shao J, Wan Q, Li L. Interimplant papilla reconstruction at second-stage surgery: a technique. J Prosthet Dent. 2022;128:554–9. 10.1016/j.prosdent.2020.09.040 33712312

[20] Sharma P, Vaish S, Sharma N, Sekhar V, Achom M, Khan F. Comparative evaluation of efficacy of subepithelial connective tissue graft versus platelet-rich fibrin membrane in surgical reconstruction of interdental papillae using Han and Takie technique: a randomized controlled clinical trial. J Indian Soc Periodontol. 2020;24:547–53. 10.4103/jisp.jisp_125_20 33424172 PMC7781246

[21] Saleh MHA, Urban IA, Alrmali A, Ravidà A. Papilla reconstruction using a vertical interproximal tunnel approach. Int J Oral Implantol. 2023;16:55–64. 36861681

[22] Pradeep AR, Karthikeyan BV. Peri-implant papilla reconstruction: realities and limitations. J Periodontol. 2006;77:534–44. 10.1902/jop.2006.050068 16512769

[23] Zanin F, Moreira MS, Pedroni ACF, Windlin M, Brugnera AP, Brugnera Júnior A, et al. Hemolasertherapy: a novel procedure for gingival papilla regeneration-case report. Photomed Laser Surg. 2018;36:221–6. 10.1089/pho.2017.4349 29652571

[24] Zachrisson BU. Interdental papilla reconstruction in adult orthodontics. World J Orthod. 2004;5:67–73. 15615145

[25] Singh VP, Uppoor AS, Nayak DG, Shah D. Black triangle dilemma and its management in esthetic dentistry. Dent Res J. 2013;10:296–301. PMC3760350 24019795

[26] McGuire MK, Scheyer ET. A randomized, double-blind, placebo-controlled study to determine the safety and efficacy of cultured and expanded autologous fibroblast injections for the treatment of interdental papillary insufficiency associated with the papilla priming procedure. J Periodontol. 2007;78:4–17. 10.1902/jop.2007.060105 17199533

[27] Ozsagir ZB, Saglam E, Sen Yilmaz B, Choukroun J, Tunali M. Injectable platelet-rich fibrin and microneedling for gingival augmentation in thin periodontal phenotype: a randomized controlled clinical trial. J Clin Periodontol 2020;47:489–99. 10.1111/jcpe.13247 31912532

[28] Neto ADT, Storrer CLM, Santos FR, Miran T. Increased papilla between implant and tooth with TEH use of hyaluronic acid injection: a case report. Int J Periodontol Implantol. 2018;3:24–9. 10.18231/2457-0087.2018.0005

[29] Yamada Y, Nakamura S, Ueda M, Ito K. Papilla regeneration by injectable stem cell therapy with regenerative medicine: long-term clinical prognosis. J Tissue Eng Regen Med. 2015;9:305–9. 10.1002/term.1737 23533047

[30] Carnio J, Carnio AT. Papilla reconstruction: Interdisciplinary consideration for clinical success. J Esthet Restor Dent. 2018;30:484–91. 10.1111/jerd.12411 30195268

[31] Jamwal D, Kanade K, Tanwar VS, Waghmare P, Landge N. Treatment of interdental papilla: a review. Galore Int J Health Sci Res. 2019;4:1–12.

[32] Fraser JR, Laurent TC, Laurent UB. Hyaluronan: its nature, distribution, functions and turnover. J Intern Med. 1997;242:27–33. 10.1046/j.1365-2796.1997.00170.x 9260563

[33] Dahiya P, Kamal R. Hyaluronic acid: a boon in periodontal therapy. N Am J Med Sci. 2013;5:309–15. 10.4103/1947-2714.112473 23814761 PMC3690787

[34] Attenello NH, Maas CS. Injectable fillers: review of material and properties. Facial Plast Surg. 2015;31:29–34. 10.1055/s-0035-1544924 25763894

[35] Fakhari A, Berkland C. Applications and emerging trends of hyaluronic acid in tissue engineering, as a dermal filler and in osteoarthritis treatment. Acta Biomater. 2013;9:7081–92. 10.1016/j.actbio.2013.03.005 23507088 PMC3669638

[36] Lee JH, Lee KE, Kang SW, Park SH, Chae YK, Lee MH, et al. Effect of orodispersible hyaluronic acid film on palatal mucosa wound healing. Oral Dis. 2023;30(2):518–27. 10.1111/odi.14517 36691707

[37] Pogrel MA, Lowe MA, Stern R. Hyaluronan (hyaluronic acid) in human saliva. Arch Oral Biol. 1996;41:667–71. 10.1016/s0003-9969(96)00050-7 9015567

[38] Zhang Y, Hong G, Zhang Y, Sasaki K, Wu H. Minimally invasive procedures for deficient interdental papillae: a review. J Esthet Restor Dent. 2020;32:463–71. 10.1111/jerd.12608 32519508

[39] Becker W, Gabitov I, Stepanov M, Kois J, Smidt A, Becker BE. Minimally invasive treatment for papillae deficiencies in the esthetic zone: a pilot study. Clin Implant Dent Relat Res. 2010;12:1–8. 10.1111/j.1708-8208.2009.00247.x 19843105

[40] Sadat Mansouri S, Ghasemi M, Salmani Z, Shams N. Clinical application of hyaluronic acid gel for reconstruction of interdental papilla at the esthetic zone. J Iran Dent Assoc. 2013;25:208–13.

[41] Bertl K, Gotfredsen K, Jensen SS, Bruckmann C, Stavropoulos A. Adverse reaction after hyaluronan injection for minimally invasive papilla volume augmentation. A report on two cases. Clin Oral Implants Res. 2017;28:871–6. 10.1111/clr.12892 27252126

[42] Higgins JPT, Thomas J, Chandler J, Cumpston M, Li T, Page MJ, et al., eds. Cochrane handbook for systematic reviews of interventions version 6.3. Cochrane; 2022 [cited 2023 Jun 20]. Available from: www.training.cochrane.org/handbook

[43] Page MJ, McKenzie JE, Bossuyt PM, Boutron I, Hoffmann TC, Mulrow CD, et al. The PRISMA 2020 statement: an updated guideline for reporting systematic reviews. BMJ. 2021;372:n71. 10.1136/bmj.n71 33782057 PMC8005924

[44] Moher D, Liberati A, Tetzlaff J, Altman DG; PRISMA Group. Preferred reporting items for systematic reviews and meta-analyses: the PRISMA statement. Int J Surg. 2010;8:336–41. 10.1016/j.ijsu.2010.02.007 20171303

[45] McHugh ML. Interrater reliability: the kappa statistic. Biochem Med. 2012;22:276–82. 10.11613/BM.2012.031 PMC3900052 23092060

[46] Sterne JAC, Savović J, Page MJ, Elbers RG, Blencowe NS, Boutron I, et al. RoB 2: a revised tool for assessing risk of bias in randomised trials. BMJ. 2019;366:l4898. 10.1136/bmj.l4898 31462531

[47] Wells GA, Shea B, O’Connell D, Peterson J, Welch V, Losos M, et al. The Newcastle-Ottawa Scale (NOS) for assessing the quality of nonrandomised studies in meta-analyses. [cited 2023 Jun 20]. Available from: https://www.ohri.ca/programs/clinical_epidemiology/oxford.asp

[48] Turgut Çankaya Z, Tamam E. An examination of the 2-year results obtained from hyaluronic acid filler injection for interdental papilla losses. Quintessence Int. 2020;51:274–84. 10.3290/j.qi.a43938 32020128

[49] Awartani FA, Tatakis DN. Interdental papilla loss: treatment by hyaluronic acid gel injection: a case series. Clin Oral Investig. 2016;20:1775–80. 10.1007/s00784-015-1677-z 26613740

[50] Firkova E. Minimally invasive reconstruction of deficient papillae with hyaluronic acid – treatment protocol and 6-months 6-results. JIMAB. 2020;26:3408–15. 10.5272/jimab.2020264.3408

[51] da Silva TZ, de Oliveira AC, Margonar R, Faeda RS, Dos Santos PL, Queiroz TP. Effectiveness of hyaluronic acid injection for interdental papillae recovery in esthetic areas: a randomized clinical trial. Int J Periodontics Restorative Dent. 2023;43:e73–80. 10.11607/prd.5814 37232687

[52] Alhabashneh R, Alomari S, Khaleel B, Qinawi H, Alzaubi M. Interdental papilla reconstruction using injectable hyaluronic acid: a 6 month prospective longitudinal clinical study. J Esthet Restor Dent. 2021;33:531–7. 10.1111/jerd.12680 33174355

[53] Pitale U, Pal PC, Thakare G, Verma M, Dhakad S, Pandey R. Minimally invasive therapy for reconstruction of lost interdental papilla by using injectable hyaluronic acid filler. J Indian Soc Periodontol. 2021;25:22–8. 10.4103/jisp.jisp_19_20 33642737 PMC7904010

[54] Patel P, Thakkar K, Kikani A, Patel V, Ravi Kiran N, Ahmed S. Minimally invasive treatment for reconstruction of deficit interdental papillae: a pilot study. J Dent Spec. 2017;5:27–30.

[55] Singh S, Vandana KL. Use of different concentrations of hyaluronic acid in interdental papillary deficiency treatment: a clinical study. J Indian Soc Periodontol. 2019;23:35–41. 10.4103/jisp.jisp_332_18 30692741 PMC6334549

[56] Ni J, Shu R, Li C. Efficacy evaluation of hyaluronic acid gel for the restoration of gingival interdental papilla defects. J Oral Maxillofac Surg. 2019;77:2467–74. 10.1016/j.joms.2019.06.190 31445036

[57] Lee WP, Seo YS, Kim HJ, Yu SJ, Kim BO. The association between radiographic embrasure morphology and interdental papilla reconstruction using injectable hyaluronic acid gel. J Periodontal Implant Sci. 2016;46:277–87. 10.5051/jpis.2016.46.4.277 27588217 PMC5005815

[58] Patil SC, Dhalkari CD, Indurkar MS. Hyaluronic acid: ray of hope for esthetically challenging black triangles: a case series. Contemp Clin Dent. 2020;11:280–4. 10.4103/ccd.ccd_42_19 33776357 PMC7989750

[59] Lee WP, Kim HJ, Yu SJ, Kim BO. Six month clinical evaluation of interdental papilla reconstruction with injectable hyaluronic acid gel using an image analysis system. J Esthet Restor Dent. 2016;28:221–30. 10.1111/jerd.12216 27159838

[60] Ebrahimi R, Khorshidi H, Boroumand R, Azadikhah A, Haddadi P. Evaluation of the effect of hyaluronic acid injection on the reconstruction of reduced interdental papillae in patients referred to shiraz school of dentistry. J Dent (Shiraz),2023;24:305–11. 10.30476/dentjods.2022.94766.1808 37727351 PMC10506141

[61] Abdelraouf SA, Dahab OA, Elbarbary A, El-Din AM, Mostafa B. Assessment of hyaluronic acid gel injection in the reconstruction of interdental papilla: a randomized clinical trial. Open Access Maced J Med Sci. 2019;7:1834–40. 10.3889/oamjms.2019.478 31316670 PMC6614259

[62] Bertl K, Gotfredsen K, Jensen SS, Bruckmann C, Stavropoulos A. Can hyaluronan injections augment deficient papillae at implant-supported crowns in the anterior maxilla? A randomized controlled clinical trial with 6 months follow-up. Clin Oral Implants Res. 2017;28:1054–61. 10.1111/clr.12917 27378556

[63] Bal A, Panda S, Mohanty R, Satpathy A, Nayak R, Tumedei M, et al. Effectiveness of hyaluronic acid gel injection with and without prgf for management of interdental papillary loss: a randomized clinical trial. J Funct Biomater. 2023;14:114. 10.3390/jfb14020114 36826913 PMC9967875

[64] Mandel I, Farkasdi S, Varga G, Nagy ÁK. Comparative evaluation of two hyaluronic acid gel products for the treatment of interdental papillary defects. Acta Stomatol Croat. 2020;54:227–37. 10.15644/asc54/3/1 33132386 PMC7586896

[65] Ni J, Zhong Z, Wu Y, Shu R, Wu Y, Li C. Hyaluronic acid vs. physiological saline for enlarging deficient gingival papillae: a randomized controlled clinical trial and an in vitro study. Ann Transl Med. 2021;9:759. 10.21037/atm-20-7599 34268372 PMC8246166

[66] Abdelkader E, Ebrahem M. Management of black triangle of interdental papilla using injectable carboxymethyl chitosan hydrogel with and without hyaluronic acid gel (Comparative Study). MJMR. 2022;33:111–20. 10.21608/mjmr.2022.251003

[67] Kapoor S, Bhardwaj A. Reconstruction of interdental papilla in esthetic zone using hyaluronic acid gel: a clinical prospective study. J Pharm Res Int. 2022;34:43–58. 10.9734/jpri/2022/v34i10B35525

[68] Fakher I, Hazzaa H, Abdelgawad N. Use of injectable hyaluronic acid gel and injectable platelet-rich fibrin in the treatment of gingival black triangles: a randomized clinical trial. AZJD. 2023;10:471–7. 10.58675/2974-4164.1510

[69] Shawky H, Darwish M. Clinical application of radiesse and hyaluronic acid gel for treatment of papillae deficiencies in the esthetic zone. EDJ. 2017;63:533–45. 10.21608/edj.2017.75003

[70] Jemt T. Regeneration of gingival papillae after single-implant treatment. Int J Periodontics Restorative Dent. 1997;17:326–33. 9497723

[71] Cardaropoli D, Re S, Corrente G. The Papilla Presence Index (PPI): a new system to assess interproximal papillary levels. Int J Periodontics Restorative Dent. 2004;24:488–92. 10.11607/prd.00.0596 15506030

[72] Chow YC, Wang HL. Factors and techniques influencing peri-implant papillae. Implant Dent. 2010;19:208–19. 10.1097/ID.0b013e3181d43bd6 20523177

[73] Fallacara A, Manfredini S, Durini E, Vertuani S. Hyaluronic acid fillers in soft tissue regeneration. Facial Plast Surg. 2017;33:87–96. 10.1055/s-0036-1597685 28226376

[74] Gonçalves Motta SH, Ferreira Camacho MP, Quintela DC, Santana RB. Relationship between clinical and histologic periodontal biotypes in humans. Int J Periodontics Restorative Dent. 2017;37:737–41. 10.11607/prd.2501 28817140

[75] Faé DS, de Aquino SN, Correa FOB, Rabelo CC, Pontes AEF, Lemos GAA. Hyaluronic acid gel as a nonsurgical approach for the interdental papillary defects: a systematic review. Dent Rev. 2023;3:100066. 10.1016/j.dentre.2023.100066

